# CROSS-NATIONAL VARIABILITY IN SEX/GENDER DIFFERENCES AND LATER-LIFE MEMORY: INDIA AND THE UNITED STATES

**DOI:** 10.1093/geroni/igac059.412

**Published:** 2022-12-20

**Authors:** Ashly Westrick, Justina Avila-Rieger, Alden Gross, Lindsay Kobayashi

**Affiliations:** University of Michigan, Ann Arbor, Michigan, United States; Columbia University Medical Center, New York, New York, United States; Johns Hopkins Bloomberg School of Public Health, Baltimore, Maryland, United States; University of Michigan, Ann Arbor, Michigan, United States

## Abstract

Little is known about the extent of sex/gender differences in later-life cognitive health in low-income countries such as India. We compared sex/gender differences in later-life memory, overall and by educational attainment, across men and women aged ≥65 years in India and the United States. Data were from Harmonized Cognitive Assessment Protocols (HCAP) in the population-representative Longitudinal Study of Aging in India (LASI; N=4,096;) and U.S. Health and Retirement Study (HRS; N=3,345). Multiple-group models estimated interactions between sex/gender and educational attainment on harmonized episodic memory scores across countries. In the U.S., women had a memory performance advantage compared to men across all education levels. In India, men had a memory performance advantage compared to women overall, interactions revealed that this advantage was only present among those with no formal education. Among those with at least lower secondary education, women demonstrated an advantage that increased with increasing education.

